# Letter to the Editor: Speedy Plant Genotyping by SDS-Tolerant Cyclodextrin-PCR

**DOI:** 10.1093/pcp/pcac093

**Published:** 2022-06-30

**Authors:** Yoichi Nakanishi, Terumi Kawashima, Mayuko Naganawa, Toshiyuki Mikami, Masayoshi Maeshima, Sumie Ishiguro

**Affiliations:** Graduate School of Bioagricultural Sciences, Nagoya University, Furo-cho, Chikusa-ku, Nagoya, 464-8601 Japan; Graduate School of Bioagricultural Sciences, Nagoya University, Furo-cho, Chikusa-ku, Nagoya, 464-8601 Japan; Graduate School of Bioagricultural Sciences, Nagoya University, Furo-cho, Chikusa-ku, Nagoya, 464-8601 Japan; Graduate School of Bioagricultural Sciences, Nagoya University, Furo-cho, Chikusa-ku, Nagoya, 464-8601 Japan; Graduate School of Bioagricultural Sciences, Nagoya University, Furo-cho, Chikusa-ku, Nagoya, 464-8601 Japan; Graduate School of Bioscience and Biotechnology, Chubu University, 1200 Matsumoto-cho, Kasugai, 487-8501 Japan; Graduate School of Bioagricultural Sciences, Nagoya University, Furo-cho, Chikusa-ku, Nagoya, 464-8601 Japan

Genotyping of genetically marked plant material is one of the most widely used methods in any field of plant research. The introduction of polymerase chain reaction (PCR) has dramatically simplified the gene determination process; however, preparing genomic DNA (gDNA) from test plants is still a time-consuming task. Careful preparation of DNA requires about 30 handling steps and ∼1 h to complete, even with a commercially available DNA purification kit. Some simple DNA purification methods from plants used only for genotyping PCR have been developed. The methods include plant disruption, DNA extraction with the detergent sodium dodecyl sulfate (SDS), purification by alcohol precipitation, drying and redissolution (Edwards et al. 1991). To improve the labor productivity, a high-throughput purification process using a 96-well format has also been reported (O’Malley et al. 2015). Aside from this, other methods have been proposed that include the DNA-extraction step but omit the steps after alcohol precipitation (Wang et al. 1993, Kasajima et al. 2004). The latter is good because there are few handling steps, but the manual details are a little tricky, so some skill is required for stable genotyping.

Here, we propose a straightforward, reproducible and time- and cost-saving genotyping method that combines hot alkaline/SDS extraction of gDNA and the direct use of the sample in a novel SDS-tolerant cyclodextrin-PCR (CD-PCR; **Fig. 1**). The preparation of gDNA is effortless: alkali has the effect of weakening the cell wall and peeling off DNA-binding proteins; the detergent SDS destroys plasma and nuclear membranes and denatures intracellular proteins to increase the DNA extraction efficiency. Such a crude extract may contain sufficient DNA for PCR template; however, it cannot usually be used for PCR because the dodecyl sulfate anion (DS^−^) caused by the dissociation of SDS inhibits the DNA polymerase in PCR. It is, therefore, necessary to remove SDS or lower the inhibitory effects of SDS by any means.

**Fig. 1:**
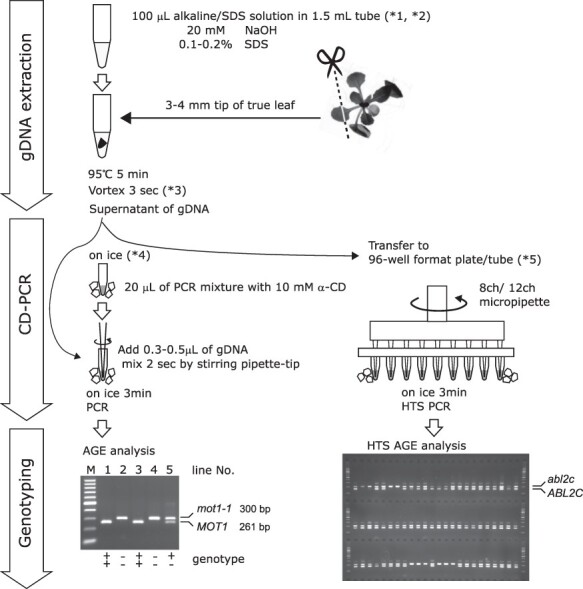
Flowchart of CD-assisted ultra-easy genotyping of plants, with examples.

Step 1. Dispense 100 μl of alkaline/SDS solution (20 mM/0.1–0.2%) into a 1.5-ml tube. Step 2. Cut 3–4 mm from the tip of an *Arabidopsis* rosette leaf and immerse in the solution. Step 3. Extract gDNA by heating the tube to 95°C for 5 min. Step 4. The supernatant is now ready for CD-PCR. Optional: Vortexing the hot tube for 3–5 s. Step 5. Optional: Transfer the gDNA sample to an 8/12/96-well format tube/plate if high-throughput screening handling is required. Step 6. The gDNA sample can be stored below −20°C until required for use in CD-PCR. Step 7. Prepare the PCR–dye mix-ture with 10 mM α-CD on ice and dispense in aliquots (usually 15–20 μl). Step 8. Add 0.3–0.6 μl of gDNA. Mix for 2 s by stirring with the pipette tip to improve PCR reproducibility, as a difference in density exists between gDNA extract and the CD-PCR mixture. Step 9. Run the PCR program, as appropriate. Step 10. Analyze the PCR products by AGE. Example 1 Genotyping of *Arabidopsis* T-DNA tagline *mot1-1*: 0.6 μl of gDNA was added to 15 μl CD–dye–PCR mixture consisting of *ExTaq* (Takara), 1× *ExTaq* buffer, 0.2 mM dNTPs, 10% sucrose, 0.01% cresol red and 10 mM α-CD and 0.25 μM three primer sets (MOT1.Fw, GATAGGCCATGGTTAGGGCTTGATGG; MOT1.Rv, CATCTTTGACGGTCCAAACTTGATGTC and SALK_LBb1, GCGTGGACCGCTTGCTGCAACT). The PCR program was as follows: 95°C for 2 min, followed by 40 cycles of 98°C for 8 s, 62°C for 20 s and 72°C for 30 s. Finally, 5 μl of the reaction product was analyzed by 1.5% AGE (left). Example 2 Genotyping of *abl2c*: 0.3 μl of gDNA was added to 15 μl homemade CD–dye–PCR mixture B1 with 0.25 μM three primer sets (pROK2_LB2_59, CTGGAACAACACTCAACCCTATCTC; ABL2C_LP2.F, GTGAATTATGGAGTAGTTGTATAAGTATGAGAAATGTTGG and ABL2C_RP2.R, CATGAATCTGTTCCAAGTGTATGTACTACCTG). The PCR program was as follows: 95°C for 2 min, followed by 40 cycles of 97°C for 10 s, 55°C for 20 s, 72°C for 60 s and then 72°C for 2 min. After the PCR, 2 μl of the reaction was analyzed by 1.5% AGE (right). Notes: *1, Alkaline/SDS solution is prepared before use from each stock solution, 1,000 mM and 10%. *2, A 1.5-ml tube with a tight snap cap (not soft-touch, not eazy-open) is recommended to prevent the tube from bursting during the heat step. *3, Vortexing the hot tube for 3–5 s and transferring it on ice improves PCR reproducibility if handling many samples at a time. Wear rubber gloves for safety. *4, Incubation on ice can be omitted if the CD-PCR mixture consists of a ‘Hot-start’ type of DNA polymerase. *5, The 96-well style gDNA extraction is not recommended for plant samples due to difficulty in handling.

## Cyclodextrin Improves PCR Tolerance to SDS

We previously invented a new technology, CD-PCR, to solve this problem (Nakanishi 2012). In this technology, SDS resistance of the DNA polymerase in PCR is improved by adding CDs, cyclic oligosaccharides that have an inclusion ability of organic compounds such as surfactants. For example, in a model experiment using *Taq* DNA polymerase, PCR without CD can tolerate only 0.003% SDS concentration, whereas adding 10 mM α-CD enables 0.05–0.1% SDS (**Supplementary Fig S1A**). CDs are synergistic with conventional technologies that enhance SDS tolerance and, therefore, can be combined with protective agents such as sucrose or betaine (**Supplementary Fig. S1B, C**). Also, CDs can be used together with commercially available tough enzymes (KOD FX, for example, Nakanishi 2012).

In this study, we further examined which CD was suitable for CD-PCR. We assumed an experiment in which a researcher brought a DNA solution containing 0.2% SDS into 1/20 volume of PCR by using a P-20 micropipette with poor accuracy, resulting in a final concentration of 0.01–0.02%. In the model experiment using *Taq* DNA polymerase, 0.02% SDS completely inhibited the reaction (**Supplementary Fig. S2**, lane 0). If appropriate CDs were added at 10 mM to the PCR mixture in advance, the PCR could be successful even in the presence of SDS. In a PCR that amplifies a 2-kb DNA fragment, α-CD made a PCR containing at least 0.06% SDS possible (**Supplementary Fig. S2**, lane 1). Also, γ-CD made the reaction feasible with at least 0.02% SDS (lane 10). Some CD derivatives had similar effects, 6-*O*-α-D-glucosyl-α-CD (lane 2), 2-hydroxyethyl-β-CD (lane 6), 2-hydroxypropyl- β-CD (lane 7), 6-*O*-α-D-glucosyl-β-CD (lane 8) and 6-*O*-α-D-maltosyl-β-CD (lane 9) upgraded PCR to have a minimum of 0.06% SDS resistance. By contrast, some CDs adversely affected the PCR. In the case of β-CD, the cheapest CD, PCR amplified other abnormally sized DNA products in addition to the target 2-kb amplicon, resulting in a smeared band pattern in agarose gel electrophoresis (AGE; **Supplementary Fig. S2**, lane 3). Methyl 1-2-β-CD (lane 4), 2,3,6-Tri-*O*-methyl-β-CD (lane 5) and cycloamylose (lane 11) showed inhibitory effects in PCR.

Next, we tested the performance of CD-PCR under more natural settings for plant genotyping (**Supplementary Fig. S3**). Similar to the model experiment described above, improvement of SDS tolerance by CDs was confirmed even in the case of PCR using complex plant gDNA as a template. In addition, it was shown that CD-PCR accepts crude plant extracts containing SDS.

## Genotyping of Plants by Combining Alkaline/SDS gDNA Extraction and CD-PCR

CD-PCR was applied to the genotyping procedure of an *Arabidopsis thaliana* T-DNA tagline mutant (**Fig. 1**). Crude gDNA was extracted from a piece of rosette leaf by hot alkaline/SDS solution (20 mM/0.1–0.2%) in the absence of any time-consuming processes, such as homogenization, cap open/close, centrifugation and pipetting. Then, 0.3–0.5 μl of the crude gDNA was directly added to 20 μl of ice-cold PCR premix consisting of DNA polymerase, buffer, dNTPs, two gene-specific primers, one vector primer, weighting agent, loading dye and 10 mM α-CD. After the thermal cycle process, PCR products were analyzed by AGE. For both *mot1-1* (a mutant T-DNA tagline of the molybdate transporter) and *abl2c* (a mutant T-DNA tagline of the anthocyanin-bluing transporter), the wild-type alleles and T-DNA-tagged mutant alleles were successfully identified by migration of allele-specific PCR products (**Fig. 1**).

The method was also tested for genotyping a tomato *jasmonic acid–insensitive1* mutant (*jai1-1*) with a deletion of about 6.5 kb in the *SlCOI1* region encoding the jasmonic acid receptor. Since homozygous plants are sterile, it is necessary to select homozygous segregants from the seed pool of heterozygous parents when using *jai1-1* for experiments (Li et al. 2004). About 3–4 mm leaf tissue was cut from the tip of a true leaf from seedlings 2 weeks after germination and then subjected to alkaline/SDS boiling gDNA extraction and CD-PCR. By using three gene-specific primers capable of distinguishing between wild-type and *jai1-1* genes, candidate plants were successfully genotyped by CD-PCR to clearly differentiate between wild-type, heterozygous and homozygous strains (**Supplementary Fig. S4**). After 2–8 weeks, the phenotype pointed out in the previous literature was confirmed in the *jai1-1* homozygous plant (Li et al. 2004); that is, suppression of jasmonic-acid-induced polyphenol oxidase activity (**Supplementary Fig. S4B**), curly leaves (**Supplementary Fig. S4C**), etc.

Finally, the genotyping of Arabidopsis seed pools in long-term storage in a dry chamber at room temperature was performed. gDNA was extracted by the alkaline/SDS boiling method from 50 seeds and analyzed by CD-PCR. Arabidopsis T-DNA tagged *vhp1-1* strain that lacks the vacuolar H^+^-pyrophosphatase gene (*VHP1*) could be distinguished from the parental Col-0, even in seed pools harvested about 20 years ago (**Supplementary Fig. S5**).

## Discussion

CDs are a class of cyclic oligosaccharides. α-CD, β-CD and γ-CD are well known, consisting of 6, 7 and 8 α-(1-4)D-glucopyranoside (Bruns 2019, Dos Santos Silva Araújo et al. 2021). CDs have a conical shape with a tunnel forming a cavity in the center, which is relatively hydrophobic in nature, while the outside displays hydrophilic properties. Various organic compounds are included in the cavity that is 0.5–0.9 nm in diameter and 0.8 nm in depth, with which CDs are able to form host–guest compound complexes. Surfactants are typical guest compounds of CDs. DS^−^ forms a CD–DS^−^ compound with a hydrophobic tail stuck in the hole of CD (Dos Santos Silva Araújo et al. 2021). A recent study reported that CD’s stoichiometry to SDS was a mixture of 2:1 and 1:1 (Ondo and Costas 2017). The thermal stability of CD-SDS inclusion is presumed to be high because the Tm of the CD inclusion compound of decanoic acid, which has a structure similar to that of the DS anion, is over 100°C (Bai et al. 2012). The protein-denaturing action of the DS^−^ occurs when its hydrophobic tail binds to the hydrophobic moiety of the protein. If CD is added to the PCR solution in advance, the hydrophobic region of DS can be masked by CD to reduce the denaturing effect and impart SDS tolerance to PCR.

α-CD is the preferred choice for CD-PCR. First because, among α, β and γ-CD, α-CD has been reported to have the highest affinity for SDS (Brocos et al. 2011). Conversely, in the case of γ-CD, *Taq* polymerase was inhibited by 0.06% SDS in PCR (**Supplementary Fig. S2** lane 10). Second, because the solubility is relatively high (149 mg/ml), a 10-fold concentration stock solution (100 mM) can be prepared. For plant genotyping, an α-CD concentration of 10 mM is sufficient (**Fig. 1**). The cost of α-CD at this concentration is relatively cheap (0.04–0.1 yen; 0.0004–0.001 US$) per 20 μl reaction. Also, a higher concentration of α-CD can be added, which results in a higher SDS tolerance (**Supplementary Fig. S6**). Some modified-β-CDs and γ-CD may be used alternatives because the spectra of organic compound inclusion may be different due to the large size of the hydrophobic cavity (Kfoury et al. 2018). When human blood was tested, hydroxyethyl-β-CD had a higher tolerance-imparting ability than α-CD (Nakanishi 2012).

It is recommended to prepare a CD-PCR-Dye premixture in which the dense solute and electrophoresis migration dye are mixed in advance so that the AGE analysis can be performed directly after the CD-PCR (**Supplementary Table S1**). A gene-specific primer pair is added to the premixture on ice immediately before use. Optionally, the CD-PCR product can be directly used for restriction enzyme reactions; our data confirm that at least some enzymes work well in such procedures (*Eco*RI, *Hae*III and *Hin*dIII, **Supplementary Fig. S7**).

The demand for PCR genotyping is increasing due to the recent development of plant molecular biology and molecular genetics using model and non-model plants. Current basic plant research involves various genetically marked tools that have been developed, for example, T-DNA-tagged deletion strains, modified endogenous genes by using genome editing technology, reporter genes, and gene expression manipulations to alter the amount, location and time patterns of genes. These genetically marked strains are combined to obtain new findings. Creating multiple mutants of homologous genes has become a standard method for reverse genetics of model plants. In this backdrop, the labor cost of PCR genotyping that is required to confirm genetic manipulation is steadily increasing. With our novel genotyping technology, researchers can drastically reduce the time and workload usually taken for PCR, thus freeing up time for more creative work in plant research.

## Supplementary Material

pcac093_Supp

## Data Availability

The data underlying this article are available in the article and in its online supplementary material. Some reference data related to minor discussion points of CD-PCR technology can be obtained from the Japanese Patent Document 2012-010666 written by Y.N.: https://www.j-platpat.inpit.go.jp/c1800/PU/JP-2012-010666/7F4C2DC16AFC3E61A46770471CB18635577C4979A24EEAA947EBD0B04D589441/11/ja.
