# Supplementary material for: Letter to the Editor: Speedy Plant Genotyping by SDS-Tolerant Cyclodextrin-PCR
Source: Plant Cell Physiol. 2022 Jun 30;63(8):1025–8. doi: 10.1093/pcp/pcac093 (PMC9381561; doi:10.1093/pcp/pcac093)
Supplement: pcac093_Supp [file pcac093_supp.zip › pcp-2022-e-00117-File003.docx]

**Materials and Methods**

*Chemicals*

α-CD, β-CD, γ-CD, 2,3,6-Tri-O-methyl-β-CD, 2-hydroxyethyl-β-CD, 6-O-α-D-Glucosyl-α-CD, 6-O-α-D-Glucosyl-β-CD were purchased from FUJIFILM Wako Pure Chemical co. Hydroxypropyl-β-cyclodextrin was purchased from Kanto chemical co. Methyl-β-CD (1.6-2.0 mol CH_3_ per unit Glc, aveMw=1310) was purchased from Sigma-Aldrich. 6-O-α-d-maltosyl-β-CD was purchased from Tokyo Chemical Industry Co., Ltd. Stock solutions of CDs were prepared at 100 mM in water (10× concentration for PCR) except for β-CD (15 mM) and 2,3,6-Tri-O-methyl-β-CD (90 mM). Reagent-grade chemicals were used except for the industry-grade detergent Mydol-12 (Kao chemical, Japan).

*Plant materials*

*Arabidopsis thaliana* T-DNA tagline *mot1-1* was kindly donated by Dr. Toru Fujiwara (Tomatsu et al., 2007). *abl2c* (SALK_050293) was obtained from Arabidopsis Biological Resource Centre (Ohio State University)*. vhp1-1* (KG8420) was obtained from Kazusa DNA Research Institute as described previously (Fukuda et al., 2016). Tomato cultivar Micro-Tom mutant with heterozygous *jai1-1/+* and its wild-type segregant (+/+) were described previously (Niwa et al., 2018).

*Enzymes*

DNA polymerases used in this study are *Taq* DNA polymerase (BioAcademia, Japan), *ExTaq* DNA polymerase (Takara Bio, Japan), QuickTaq HS Dye-Mix (Toyobo, Japan), and KOD FX Neo (Toyobo, Japan).

*Preparation of DNA polymerase to make cheap PCR-dye premix*

*E. coli* expression vector for *Taq* DNA polymerase (pAKTaq) and *Pfu* DNA polymerase (pET16B.Pfu) were obtained from AddGene (#25712, #12509).

Laboratory *Taq* DNA polymerase was purified from *E. coli* DH5a pAKTaq cultured in LB medium supplemented with ampicillin and 0.5 mM IPTG. *E. coli* pellet was suspended by 10-fold volume of buffer A (30 mM Tris-HCl, pH7.9, 30 mM KCl, 1 mM EDTA, 0.2 mg/mL lysozyme), incubated at 37°C for 10m, then the cell was disrupted by adding final 1 mM DTT, 1 mM PMSF and 0.25% Mydol-12. The extract was incubated at 77°C for 1 h and centrifuged at 10,000 rpm at 4°C for 20 m. The supernatant was applied to NucleoBond AX column (Macherey-Nagel, DNA grade anion-exchange column), washed by buffer D (20 mM Tris-HCl, pH 7.5, 0.1 mM EDTA-Na_3_, 0.1% Mydol-12, 1 mM DTT, 0.5 mM PMSF, 40 mM KCl), then eluted by buffer E (buffer D with KCl increasing to 200 mM). The peak fractions were collected, adjusted to a storage condition (5 U/μL enzyme, 10 mM Tris-HCl, pH7.5, 0.1 mM EDTA-Na_3_, 0.1% Mydol-12, 1 mM DTT, 50 mM KCl, 50%(v/v) glycerol), then stored at -20°C for 1-month storage or at -80°C for long-term storage.

Homemade *Pfu* DNA polymerase was purified from a pellet of *E. coli* BLR (DE3) pET16B.Pfu that was cultured in an autoinduction medium (2× LB Lennox, 20 mM Tris, 10 mM Mops, 1 mM MgSO_4_, 0.5% glycerol, 0.05% glucose, 0.2% lactose, 50 mg/L ampicillin) at 30°C for 23h. The bacterial pellet was suspended by ten-volume buffer S (25 mM Tris-HCl, pH 7.9, 25 mM KCl, 1 mM EDTA-Na_3_, 200 mM sorbitol, 1 mM DTT, 0.1 mg/mL lysozyme), incubated 37°C for 10 min, extracted by addition of 0.1% Mydol-12. The extract was incubated at 95°C for 5 min, then centrifuged at 15,000 rpm for 5 min. The supernatant was applied to Ni-NTA agarose (Qiagen) column chromatography for purification. The column was washed twice with ten-volume wash buffer (25 mM Tris-HCl, pH7.9, 100 mM KCl, 0.1% Mydol-12, 10 mM imidazole), and eluted by a similar buffer containing 300 mM imidazole. The elute fraction was adjusted for storage condition (0.5× elution buffer, 0.5 mM DTT, 0.5 mM EDTA, 50%(v/v) glycerol) then stored at -20°C until use. The activities of homemade *Taq* and *Pfu* above were determined by PCR.

*Genomic DNA purification*

Purified genomic DNA for model experiments was prepared from yeast *Ogataea polymorpha* JCM3620 or *Arabidopsis thaliana* Col-0 using ISOPLANT II Kit (Nippon gene, Japan), followed by RNase A treatment in TE buffer for 30 min, twice phenol/chloroform extraction, and ethanol precipitation. The concentration of the purified DNA was measured by the H33258 fluorescence method.

**Supplementary Tables**

**Table S1. Examples of CD-PCR-Dye premix**

CD-PCR-dye premixtures without primer DNA were prepared on ice from stock solutions: 10× PCR buffers, 2.5 mM dNTP, 100 mM CDs, 60% sucrose, and 1% dyes with gentle agitation. The premix was dispensed into polypropylene microtubes and cryopreserved until use. The mixtures withstand at least ten freeze-thaw cycles. *Relatively high concentrations were used because the activity value of the homemade enzyme has more variation than that of a commercial-grade enzyme.

*Taq* base mix *ExTaq* base QuickTaq base Home-made “B1”

buffer 1× *Taq* buffer 1× *ExTaq* buffer (not disclosed) 60 mM Tris-HCl (8.8)

20 mM (NH_4_)_2_ SO_4_

2 mM MgSO_4_

substrate 0.2 mM dNTP 0.2 mM dNTP (not disclosed) 0.2 mM dNTP

cyclodextrins 10 mM α-CD 10 mM α-CD 10 mM α-CD 10 mM α-CD

3 mM glucosyl-β-CD

enzyme 0.025 U/μL *Taq* 0.025 U/μL *ExTaq* 1× QuickTaq *0.04 U/μL *Taq*

(Any maker) (TaKaRa) HS DyeMix *0.0025 U/μL *Pfu*

(Toyobo)

dense solute 12% sucrose 12% sucrose - 11% sucrose

dye 0.005% cresol red-Na 0.01% orange G - 0.005% bromophenol blue

**Supplementary Figures**

**
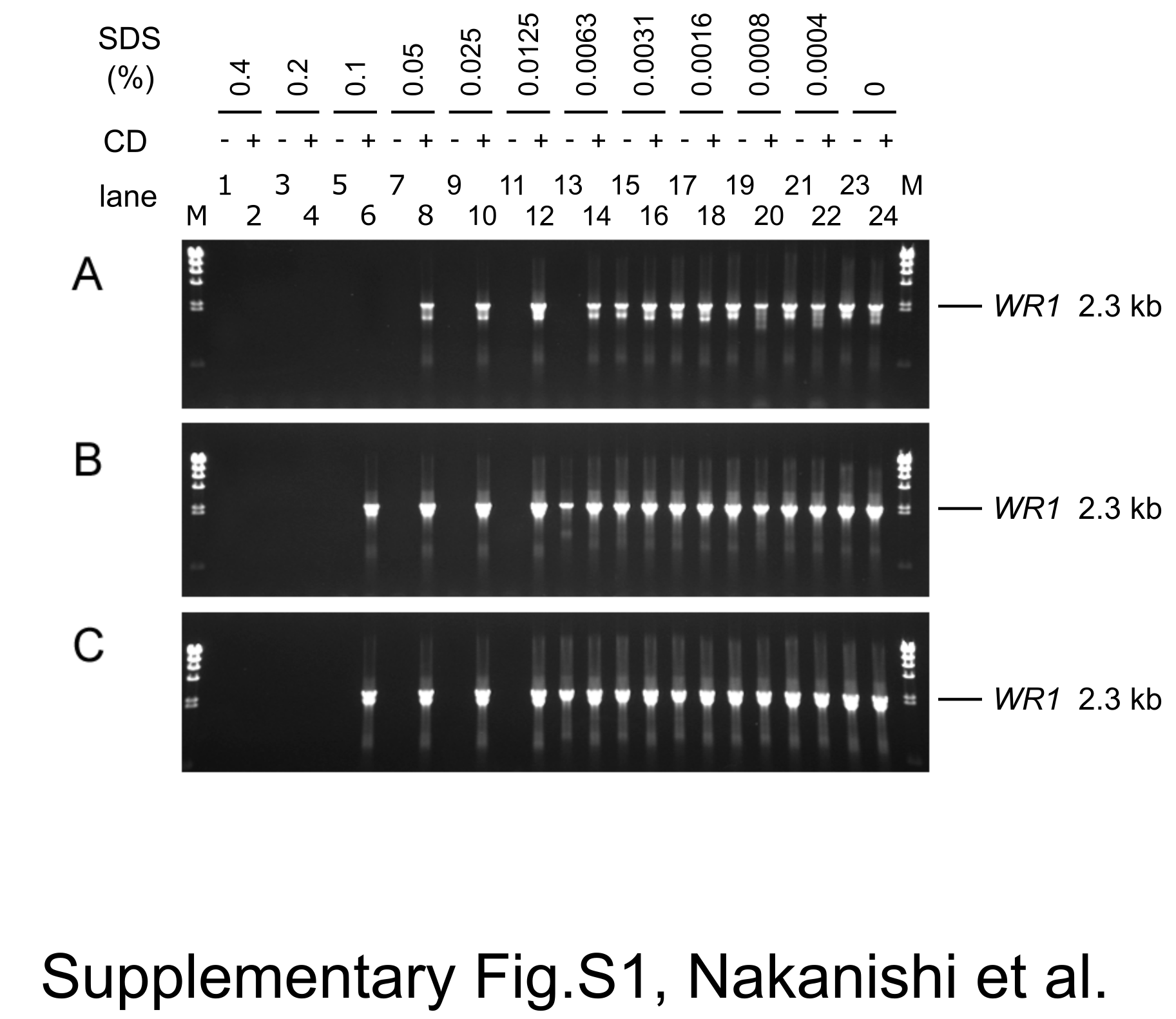
**

**Supplementary Figure S1. α-Cyclodextrin and other protective agents synergetically protect PCR reaction against SDS**

PCR for amplifying 2.3 kbp of the yeast *Ogataea polymorpha* (=*Hansenula polymorpha*) *WR1* gene was performed in the presence of SDS, α-CD, and conventional protective agents. **A**, Premixture consists of 0.025 U/μL *Taq* DNA polymerase (BioAcademia, Osaka, Japan), 1× reaction buffer, 0.2 mM dNTPs, 0.02 ng/μL of purified gDNA from yeast *Ogataea polymorpha* JCM3620, 0.25 μM each primer pair (WR1-228Fw, GGCGATATACATTGAAAGGTAAATAAGGG; wr1-96rv, GTCTTCACCTCTGAATATTGGCCGT) supplemented with 10 mM α-CD was prepared on ice (CD +). Then, the PCR composition was mixed with SDS of 0 to 0.4% at the final concentration and kept on ice for 2 m. The thermal process of PCR was as follows: 94°C 5 m, followed by 35 cycles of (95°C 20 s, 60°C 20 s, 72°C 2 m 30 s), 72°C 2 m. The PCR reaction without α-CD was also performed (CD -). The PCR products were analyzed by 0.7% agarose gel electrophoresis: lane M, molecular weight marker. **B**, The same PCR reaction as A was performed with the addition of 12% (w/v) sucrose. **C**, The same PCR reaction as A with the addition of 1M betaine.

(This figure is a citation from the YN patent figure 2. Reprint permission by Nagoya University.)


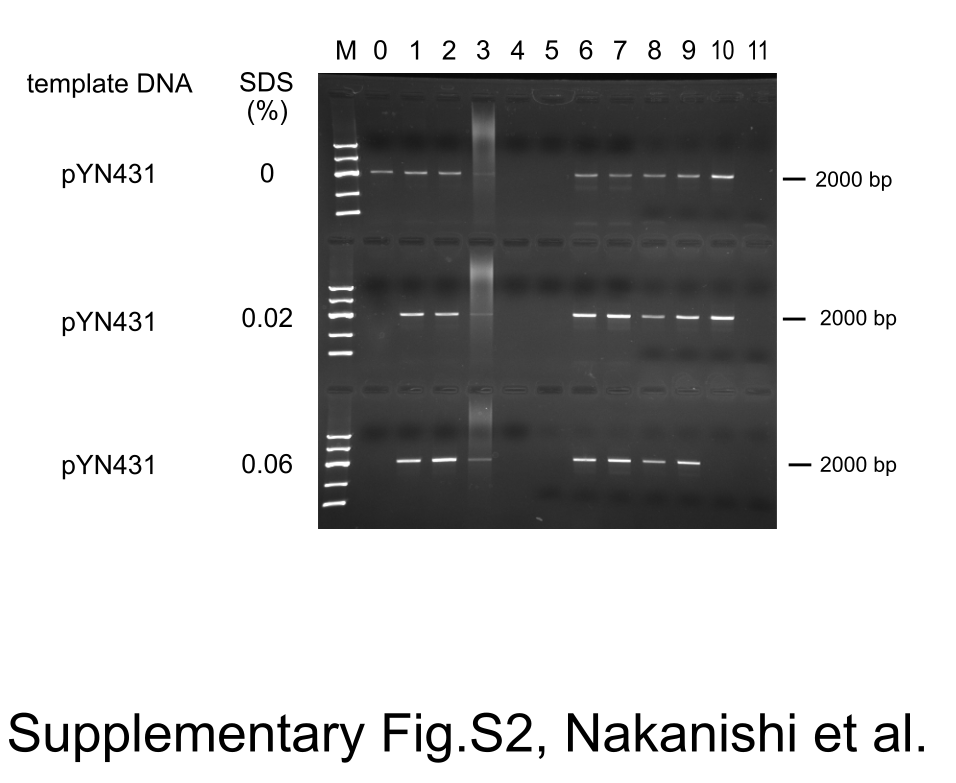


**Supplementary Figure S2. Effect of SDS and cyclodextrins on PCR reaction**

PCR for amplifying a 2000 bp amplicon was performed in the presence of SDS and cyclodextrins. Premixture consist of 0.025 U/μL *Taq* DNA polymerase (BioAcademia, Osaka, Japan), 1× reaction buffer, 0.2 mM dNTPs, 10^4^ copies of template pYN431, 0.25 μM each primer pair (pYN431_10501.F, CTTCCGGTCAAACGTCCGAT; pYN431_12500.R, TTTGAGAACGACAGCGACTT) supplemented with 10 mM CDs were prepared on ice. Then, the PCR composition was mixed with final SDS concentrations of 0, 0.02, 0.06%, and kept on ice for 2 m. The thermal process of PCR was as follows: 94°C 2 m, followed by 30 cycles of (95°C 20 s, 53°C 20 s, 72°C 2 m), 72°C 2 m. The PCR products were analyzed by 0.7% agarose gel electrophoresis. Lane M, molecular weight marker; lane 0, without CDs; lane 1, α-CD; lane 2, 6-O-α-D-Glucosyl-α-CD; lane 3, β-CD; lane 4, Methyl_1.6-2_-β-CD; lane 5, 2,3,6-Tri-O-methyl-β-CD; lane 6, 2-hydroxyethyl-β-CD; lane 7, 2-hydroxypropyl-β-CD; lane 8, 6-O-α-D-Glucosyl-β-CD; lane 9, 6-O-α-D-maltosyl-β-CD; lane 10, γ-CD; lane 11, cycloamylose (22-50 glucose unit).

**
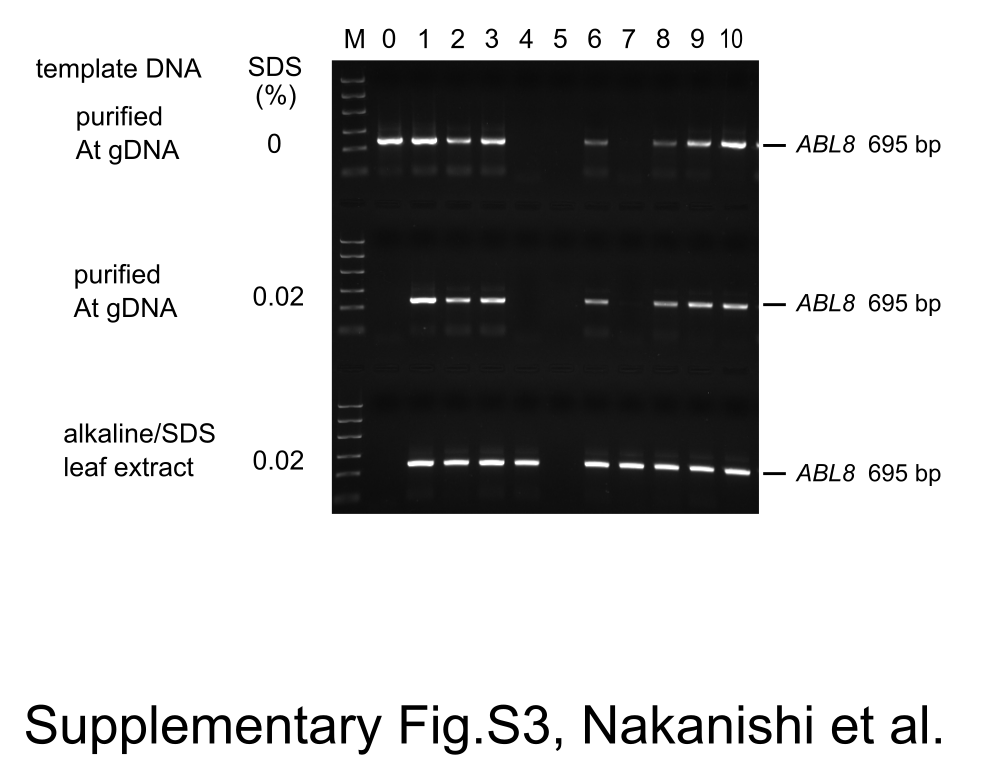
**

**Supplementary Figure S3. Cyclodextrins enable PCR of plant DNA samples containing SDS**

PCR for amplifying the *Arabidopsis thaliana* *ABL8* gene was performed using plant genomic DNA as a template in the presence of SDS and/or crude extract. Premixture consist of 0.025 U/μL *Taq* DNA polymerase (BioAcademia, Osaka, Japan), 1× reaction buffer, 0.2 mM dNTPs, 0.25 μM primer pair (ABL8-47.L, GGGAACGGTACGTTTACTACTTCTTCTTTG; ABL8-47.R, CATAACGAAAGTGAGGGAAGGCAT-TATGG) supplemented with 10 mM CDs were prepared on ice. Then, the PCR composition was mixed with a template DNA sample and kept on ice for 2 m. The template DNA samples were: upper row, 1.6 pg/μL purified gDNA from *A.thaliana* Col-0; middle row, the gDNA supplemented with 0.02% SDS; lower row, 10%v/v alkaline/SDS extract prepared from *A.thaliana* Col-0 true-leaf as described in Fig.1 (containing 1 mM NaOH and 0.02% SDS). The thermal process of PCR was as follows: 94°C 2 min, followed by 40 cycles of (95°C 20 s, 53°C 20 s, 72°C 2 m), 72°C 2 m. The PCR products (695 bp amplicon) were analyzed by 0.7% agarose gel electrophoresis. Lane M, molecular weight marker; lane 0, without CDs; lane 1, α-CD; lane 2, 6-O-α-D-Glucosyl-α-CD; lane 3, β-CD; lane 4, Methyl_1.6-2_-β-CD; lane 5, 2,3,6-Tri-O-methyl-β-CD; lane 6, 2-hydroxyethyl-β-CD; lane 7, 2-hydroxypropyl-β-CD; lane 8, 6-O-α-D-Glucosyl-β-CD; lane 9, 6-O-α-D-maltosyl-β-CD; lane 10, γ-CD.

**
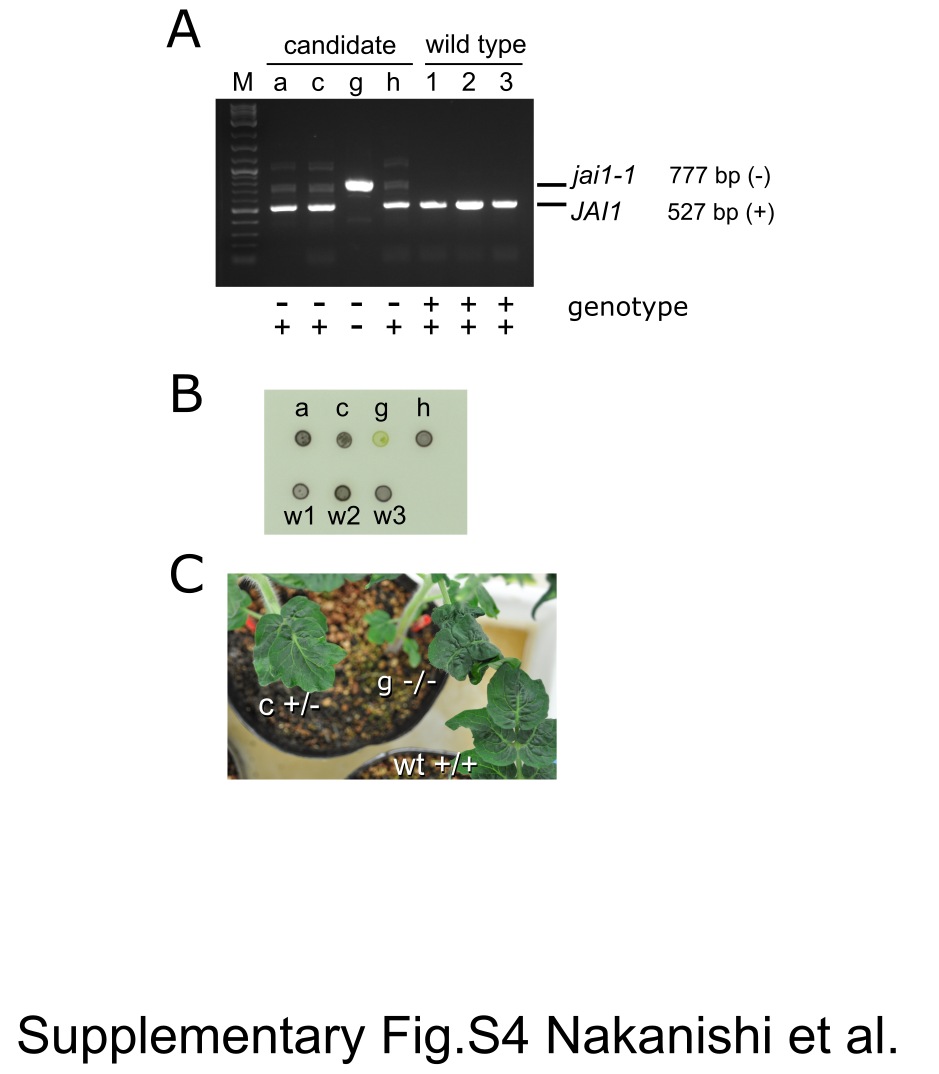
**

**Supplementary Figure S4. Genotyping of Micro-Tom**

(A) Genotyping of tomato. gDNA was extracted by alkaline/SDS (20 mM/0.1%) from true-leaf of 2 weeks-old *ja1-1* candidates (a, c, g, h) or wild-type (1, 2, 3) Micro-Tom plants. 0.3 μL of the gDNA was mixed with 20 μL of genotyping CD-PCR mixture consisting of the homemade *Taq-Pfu*-dye mixture (B1) and three gene-specific primers (LeCoi1.1524F, GTGGAGACGATATGTTGAGACTAAGTG; LeCoi1.2050R, CCATGGAGTCCATCACCTAACAGT; LeCoi1.8543R, GTGGTCAGATCAGAGCCCTCTATT). PCR was performed with an initial denaturation step at 95°C 2 m followed by 40 cycles of (98°C 10 s, 55°C 20 s, 72°C 60 s), 72°C 2 m. 5 μL of the PCR product was analyzed by 1.5% AGE. Genotypes were judged from the migration of *JAI1* and *jai1-1* specific PCR products. (B) *JAI1*-dependent MeJA-induced polyphenol oxidase (PPO) activity. Cut leave of candidate tomato was treated MeJA for 2days. Crude extract from the leaves was spotted on a nitrocellulose membrane. PPO activity was visualized as described previously with a few modifications (Howe and Ryan, 1999). (C) Curing leaf phenotype of *jai1-1* homozygous plant. The picture shows the top view of 4 weeks-old plants, leaf of heterozygous line c (+/-), homozygous g (-/-), and control wild-type (+/+)*.*


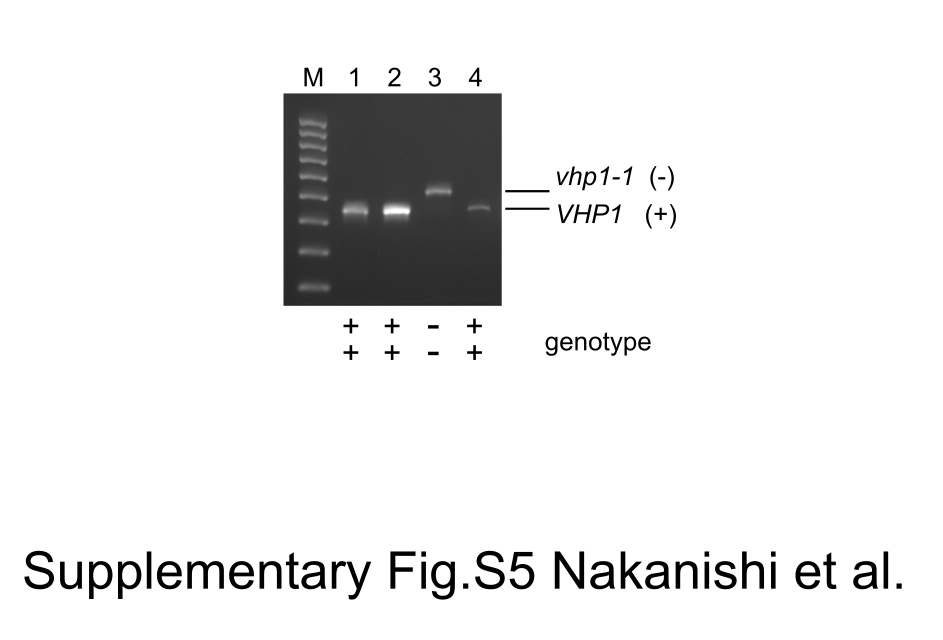


**Supplementary Figure S5. Genotyping of *Arabidopsis thaliana* seed pools stored for long periods**

About 50 grains of *Arabidopsis* seed were measured by seed-spoon 50 (Biomedical Science, Tokyo, Japan). gDNA was extracted by 200 μL of alkaline/SDS solution (20 mM, 0.2%) for 10 min at 95°C. Then 0.5 μL of the gDNA was added to PCR mixture consists of 1× QuickTaq HS DyeMix, 10 mM α-CD, and 0.25 μM primer trio (VHP1.2450F, CATCCCTGGACTTATGGAAGGAACC; VHP1.2887R, TTTGCGTGCTCTGATACAC-CAGC; P06LB, AAGAAAATGCCGATACTTCATTGGC). PCR was performed with an initial denaturation step at 95°C 2 m followed by 40 cycles of (98°C 10 s, 60°C 20 s, 72°C 45 s), then 5 μL of PCR product was analyzed by 1.5% AGE. Samples are 9 years old wild-type Col-0 seeds (lane 1) and 20 years old seed pools (Col-0, lane 2; homozygous *vhp1-1*, lane 3; wild-type segregant from *vhp1-1/+*, lane 4).


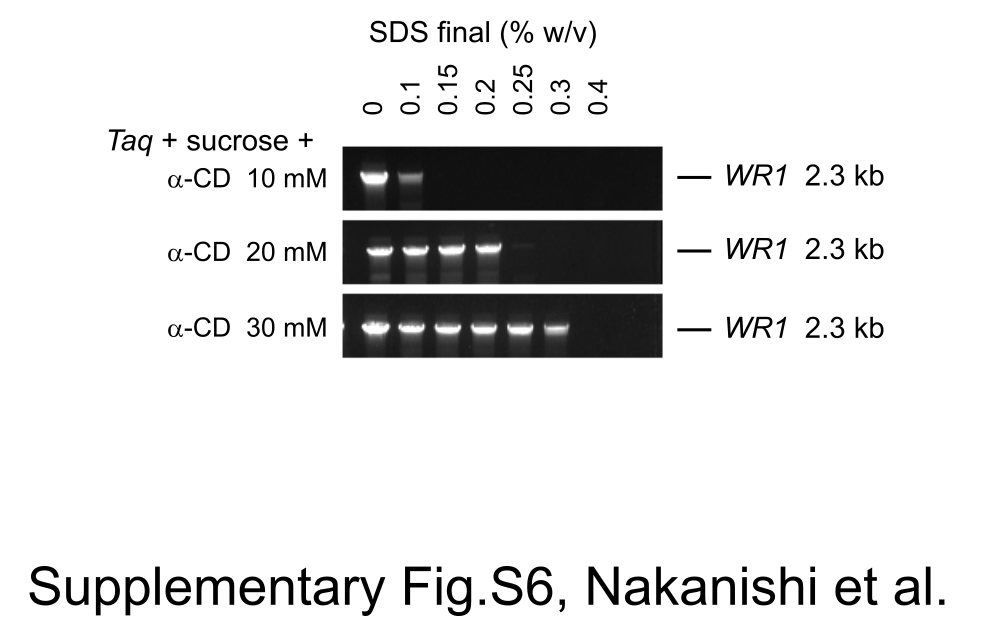


**Supplementary Figure S6. Increasing α-cyclodextrin concentration enhances PCR SDS resistance**

Cyclodextrin-PCR for amplifying 2.3 kbp amplicon for the *Ogataea polymorpha* *WR1* gene was investigated with higher concentrations of α-CD. CD-PCR premixtures were prepared to contain 12%w/v sucrose and α-CD (10, 20, and 30 mM final concentration). Then, the PCR premixture was mixed with SDS to obtain a final concentration of 0 to 0.4%. PCR thermal cycle and agarose gel analysis were as identical to Figure S1.

(This figure is a citation from the YN patent figure 4. Reprint permission by Nagoya University.)

**
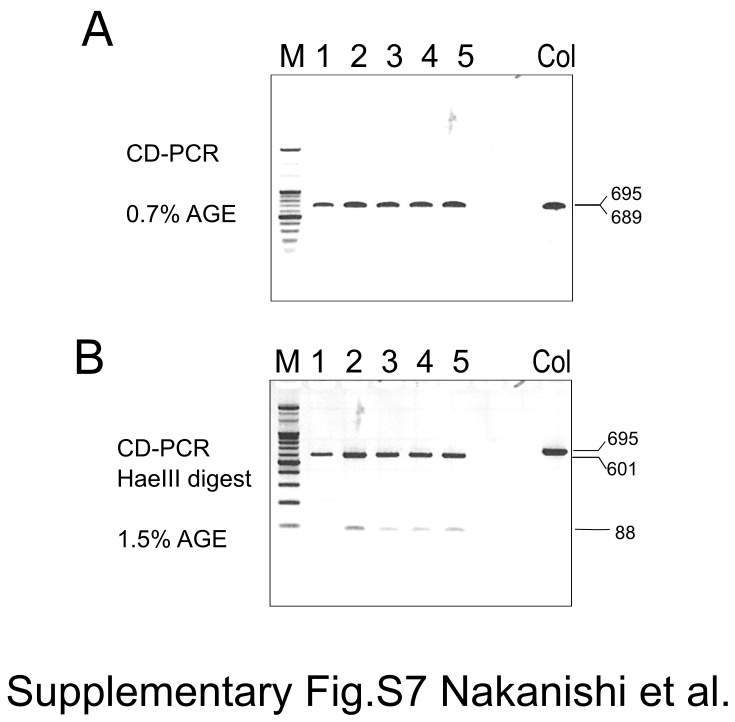
**

**Supplementary Figure S7. Application of CD-PCR product for restriction fragment length analysis**

gDNA was extracted from true-leaf of 2 weeks-old *Arabidopsis* T-DNA tagged *ame-0302* candidates (lane 1-5) or a parental wild-type plant (Col) by alkaline/SDS (20 mM/0.1%) method. 0.3 μL of the crude gDNA was mixed with 15 μL of CD-PCR mixture containing 0.02 U/μL KOD FX Neo DNA polymerase (Toyobo), 1× KOD FX Neo reaction buffer, 0.4 mM dNTPs, 10 mM α-CD, 0.2 μM each primer pairs for *AME-0302 gene* (A0302.F, GCAAAGCCTTCTGAACCAAT; A0302.R, GCCGGATCCACCGTCAACATGTTCATGAGA-AGAATCAATG) and 0.2 μM T-DNA-specific primer (LBb1.3, ATTTTGCCGATTTCGGAAC). PCR was performed with an initial denaturation step at 95°C 3 m followed by 40 cycles of (97°C 10 s, 55°C 20 s, 68°C 1 m 20 s), 68°C 2 m. (A) 3 μL of the PCR product was analyzed by 0.7% AGE. Due to the comparable size of the PCR products (wild-type gene 695 bp; tagged-gene 689 bp), the genotype cannot be distinguished from the results. (B) Restriction enzyme-digestion fragment length analysis of the PCR product. 3 μL of the PCR product was mixed with 7 μL of the restriction enzyme premix containing 0.2U/μL HaeIII, 1.4× M buffer (Takara), 8% sucrose, and 0.01% cresol-red (gel loading pigment). After incubation at 37°C for 2 h, the obtained DNA fragment was analyzed by 1.5% AGE. Only PCR product from the T-DNA-tagged *ame-0302* gene was cleaved by HaeIII, yielding 601 bp and 88 bp fragments.

**Supplementary Reference**

Fukuda, M., Segami, S., Tomoyama, T., Asaoka, M., Nakanishi, Y., Gunji, S., et al. (2016) Lack of H+-pyrophosphatase Prompts Developmental Damage in Arabidopsis Leaves on Ammonia-Free Culture Medium. *Frontiers in Plant Science*. 7.

Howe, G.A., and Ryan, C.A. (1999) Suppressors of Systemin Signaling Identify Genes in the Tomato Wound Response Pathway. *Genetics*. 153: 1411–1421.

Niwa, T., Suzuki, T., Takebayashi, Y., Ishiguro, R., Higashiyama, T., Sakakibara, H., et al. (2018) Jasmonic acid facilitates flower opening and floral organ development through the upregulated expression of SlMYB21 transcription factor in tomato. *Bioscience, Biotechnology, and Biochemistry*. 82: 292–303.

Tomatsu, H., Takano, J., Takahashi, H., Watanabe-Takahashi, A., Shibagaki, N., and Fujiwara, T. (2007) An Arabidopsis thaliana high-affinity molybdate transporter required for efficient uptake of molybdate from soil. *Proceedings of the National Academy of Sciences*. 104: 18807–18812.
